# The metastasis suppressor protein NM23-H1 modulates the PI3K-AKT axis through interaction with the p110α catalytic subunit

**DOI:** 10.1038/s41389-021-00326-x

**Published:** 2021-04-30

**Authors:** Francesco Paolo Pennino, Masanao Murakami, Massimo Zollo, Erle S. Robertson

**Affiliations:** 1 Department of Otorhinolaryngology-Head and Neck Surgery, Perelman School of Medicine, Tumor Virology Program, Abramson Cancer Center, University of Pennsylvania, Philadelphia, PA USA; 2 Health Sciences Department of Medical Laboratory Science, Kochi Gakuen University, Nankoku, Kochi Japan; 3 Dipartimento di Medicina Molecolare e Biotecnologie Mediche DMMBM, Universita‘ di Napoli Federico II, Naples, Italy; 4 CEINGE Biotecnologie Avanzate, Naples, Italy; 5 European School of Molecular Medicine, SEMM, University of Milan, Milan, Italy; 6 Tumor Virology Program, Abramson Cancer Center, University of Pennsylvania, Philadelphia, PA USA

**Keywords:** Metastasis, Molecular biology

## Abstract

The PI3K pathway is one of the most deregulated pathways in cancer, which is predominantly due to gain of function mutations or altered expression of the *PI3KCA* gene. This is codified by what is seen for the class I PI3K catalytic subunit p110α, a common feature of many cancers. The metastasis suppressor protein NM23-H1 (NME1), whose ability to suppress the metastasis activities of different tumors has been widely described and was previously reported to alter phosphatidylinositol signaling. Here, we show interaction of NM23-H1 with the p110α subunit and the functional consequence of this interaction. This interaction is predominantly localized at the plasma membrane with some signals seen in the cytoplasmic compartment. Analysis of NM23-H1 levels showed a negative correlation between NM23-H1 expression and Akt phosphorylation, the key marker of PI3K pathway activation. Investigating the functional consequence of this interaction using cell motility and clonogenicity assays showed that expression of *NM23-H1* reversed the enhanced migration, invasion, adhesion, and filopodia structure formation in cells expressing the p110α catalytic subunit. A similar trend was seen in anchorage-independent assays. Notably, differential analyses using NM23-H1 mutants which lacked the enzymatic and metastasis suppressor activity, showed no detectable interaction between p110α and the NM23-H1 mutant proteins P96S, H118F, and S120G, as well as no dysregulation of the PI3K-AKT axis.

## Introduction

Cancer progression is a dynamic process that reaches its peak with the formation of a metastatic clone at a different position from the primary site. In this context, the *NM23-H1* (*NME1*) gene was shown to be a negative “Master Regulator” of cancer cells motility. This occurs either through multiple enzyme activities (nucleoside diphosphate kinase (NDPK), protein histidine kinase, and a serine/threonine-specific protein kinase 3′-5′ exonuclease activity), or through its extensive interactome, that negatively regulates signaling pathways which leads to metastasis. NM23-H1 was characterized as a metastasis suppressor by Steeg et al.^1^ during an analysis of genes differentially expressed in the murine melanoma cell line (K1735), where highly metastatic clones showed the lowest NM23-H1 expression^1^. The functional characterization of different NM23-H1 protein mutants, which include P96S, a site corresponding to the k-pn motif responsible for developmental defects in Drosophila, exhibited normal autophosphorylation and nucleoside-diphosphate kinase (NDPK), but is deficient in phosphotransfer activity. The H118F mutation lies within a site crucial for NDPK and histidine protein kinase activity, and the S120G mutation which is found in aggressive Human Neuroblastoma retains NDPK activity, but lacks histidine dependent serine phosphorylation, link the anti-motility activity of NM23-H1 to its enzymatic activity. However, it was shown that the P96S and S120G mutations showed a reduced hexameric and increased dimeric oligomerization compared to the wild type. This has been associated with a reduction in colonization and invasion suggesting that the metastasis-suppressor activity of NM23-H1 may depend on its oligomeric structure, which can affect its enzymatic activities, as well as its protein-protein interaction network^2–4^.

The Class I phosphoinositide 3-kinases consist of four different p110 catalytic subunits, -α-β, -ɣ, and -δ and only this class is involved in production of Phosphatidylinositol 3-phosphate (PtdIns3*P* or PI3P)^5^. The p110α and p85α are the most characterized PI3K catalytic and regulatory subunits, respectively, because of their association with cancer progression and tumorigenesis while the other subunits are rarely associated with tumorigenesis^6^. Production of PI3P which initiates from PI2P is the main function of the p110α kinase^7^. This process is a starting point for recruitment of effector proteins which contain a Pleckstrin Homology Domain (or PHD). Among these proteins, PDK1 (3-phosphoinositide-dependent kinase 1) and its binding partner, the serine-threonine kinase Akt are the main effectors. After PI3P-PDK1 mediated activation, Akt is fully activated by phosphorylation at Ser473 and Thr 308 thus activating the signaling cascade^8^. Indeed, *gain of function* mutations of the p110α gene (*PI3KCA*) occurs in up to one-third of human colorectal cancers (CRCs). The most frequent p110α cancer-specific *gain of function* mutation is the H1047R mutation and is responsible for increased migration, filopodia formation, and significant changes in cell morphology^9^. Notably, Qian et al. have shown that activation of PI3K alone is sufficient to remodel actin filaments and increase cell migration through the activation of protein kinase B (PKB) or Akt^10^. This preliminary characterization of the interaction between NM23-H1 and the p110α kinase puts forward an interesting scenario towards understanding the complex mechanism that underlying the anti-metastatic behavior of the NM23-H1 protein.

## Results

### NM23-H1 interacts with the p110α catalytic subunit of PI3K

Previous studies have shown that NM23-H1 was associated with signaling activities that are linked to kinase activation at the cell membrane^11^. Therefore, we asked whether or not PI3K is regulated by NM23-H1 resulting in changes in its downstream activities affecting cell migration. We thus examined the interaction of NM23-H1 with the two subunits of PI3K.

**Fig. 1:**
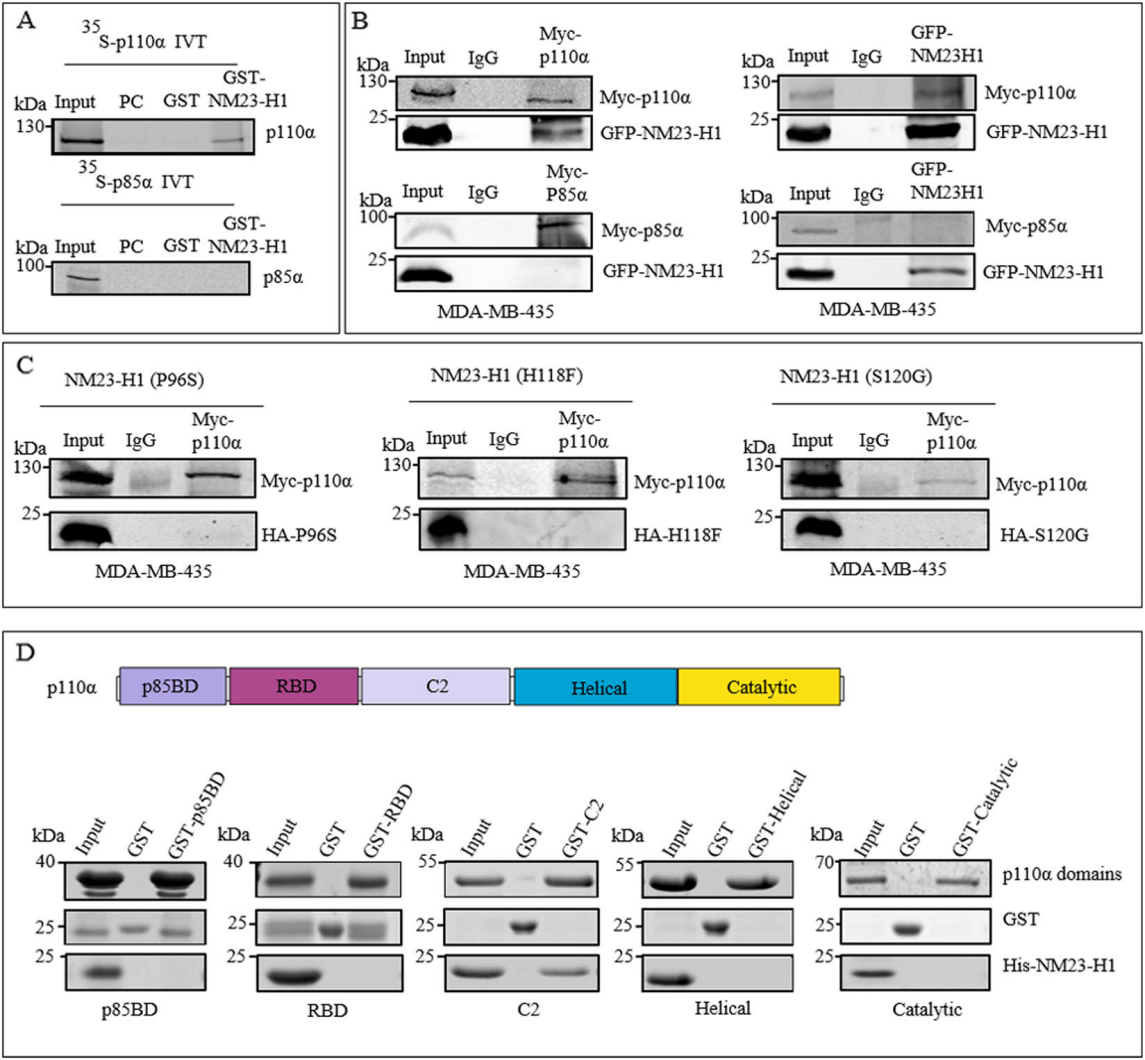
The p110α catalytic subunit of PI3K interacts with NM23‐H1 in vitro and forms a protein-protein complex in cells. **A** [^35^S] methionine‐labeled in vitro translated p110α and p85α were incubated with either GST or GST‐NM23‐H1 fusion protein. Bound proteins were resolved on SDS‐PAGE and analyzed using Image Quant software. Lane 1, 5% input labeled lysate; lane 2 pre-clear, lane 3 pull-downs with GST; lane 4, pull-down with GST‐NM23‐H1 fusion protein. **B** Cell lysates from MDA-MB-435 cells transfected with Myc-p110α (upper panel) or p85α (bottom panel) plasmids were incubated for Co-IP with either IgG, Anti-Myc antibody or Anti-GFP antibody. Bound proteins were resolved on SDS‐PAGE and membranes were analyzed by the Odyssey imaging system. Lane 1, 5% MDA-MB435 cell lysates; lane 2, Co-IP with Mouse IgG; lane 3, Co-IP with Myc fusion protein (left panel) or GFP fusion protein (right panel). **C** Cell lysates from MDA-MB-435 transfected with Myc-p110α and HA-P96S, HA-H118F or HA-S120G NM23-H1 mutant proteins were resolved on SDS‐PAGE and membranes were analyzed by the LI-COR Odyssey imaging system. Lane 1, 5% MDA-MB-435 cell lysates; lane 2, Co-IP with mouse IgG; lane 3, Co-IP with Myc-p110α fusion protein. **D** In vitro GST-pull down analysis of different domains of p110α (p85BD, RBD, C2, helical, and catalytic domains) with His-NM23-H1. The C2 domain of p110α interacts with NM23-H1.

PI3K exists as a heterodimer containing a regulatory subunit, known as p85, and a catalytic subunit, known as p110α^5^. A GST fusion protein of NM23-H1 (GST-NM23-H1) was generated and incubated with in vitro-transcribed/translated ^35^S-labeled p85α or p110α. While a GST control protein was unable to bind either p85α or p110α, GST-NM23-H1 bound only to p110α (Fig. 1A). To further support our findings, co-immunoprecipitation (Co-IP) assays using MDA-MB-435 cell lysates overexpressing Myc-p110α, Myc-p85α, and GFP-NM23-H1 was performed in both directions. The Western blot assays for detection of p85α did not show any detectable binding to GFP-NM23-H1 (Fig. 1B, lower panel). However, p110α showed complex formation with GFP-NM23-H1 (Fig. 1B, upper panel). To further investigate the association of NM23-H1 with p110α in cells we tested the NM23-H1 mutants P96S, H118F, S120G, which lack the anti-motility activity of the WT isoform^2–4^. Our Co-IP assays showed that these NM23-H1 mutants were not able to form complexes with p110α in MDA-MB-435 cell lysates which expressed the Myc-p110α, and the HA-P96S, HA-H118F nor the HA-S120G mutant. These results suggest that the amino acid residues involved in NM23-H1 enzymatic and anti-motility activities are potentially crucial for this interaction (Fig. 1C). The p110α subunit consist of five domains (p85-binding domain (p85BD), Ras-binding domain (RBD), C2, helical, and catalytic domains). We generated GST-fusions with these five domains and performed in vitro binding assays using the GST-tagged proteins, which encompassed the five domains of p110α. Our results showed that the C2 domain was responsible for the interaction of p110α with NM23-H1 (Fig. 1D).

### NM23-H1 co-localized with p110α at the cellular membrane

**Fig. 2:**
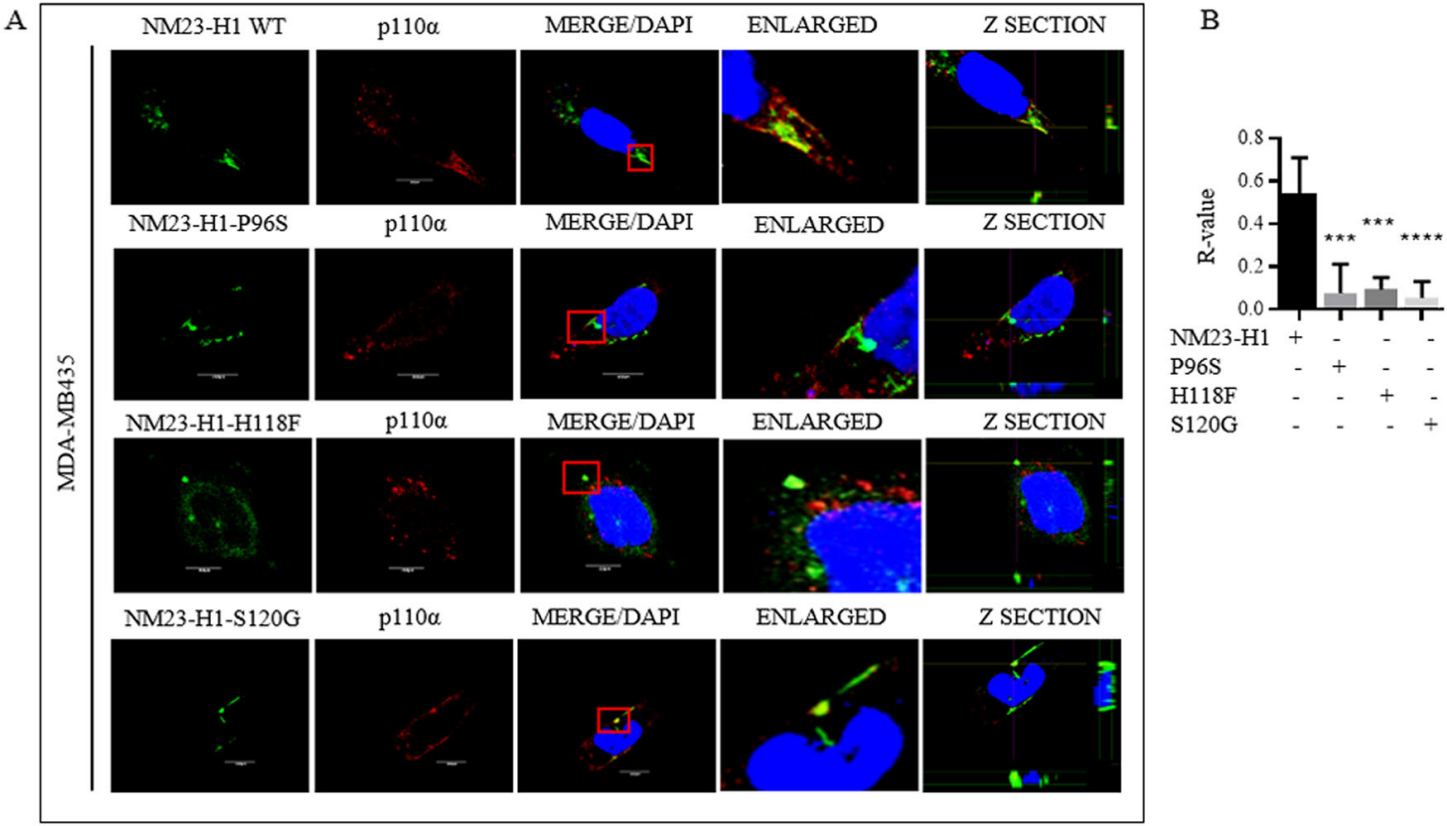
p110α co-localizes with NM23‐H1 in MDA-MB-435 cell lines. **A** Cells were transfected with both HA-NM23‐H1 and Myc-p110α, or the NM23-H1 mutated isoforms HA-P96S, HA-H118F, HA-S120G constructs. Cells were fixed in 4% PFA and screened for NM23-H1 and p110α signals using the anti-HA antibody and anti-p110α antibody, respectively. Proteins were detected with the green channel for NM23‐H1, P96S, H118F, S120G, and the red channel for p110α using a Fluoview confocal microscope. Nuclei were stained with 4′,6′‐diamidino‐2‐phenylindole (DAPI, blue). Merged images were composed from three independently acquired images. Scale bar 10 µm. **B** *R*-value co-localization score was measured with ImageJ-NIH software (*p < 0.05).

Immunofluorescence assays to determine localization of NM23-H1 and p110α showed that NM23-H1 signals was distributed in almost all cell compartments with a predominant cytoplasmic localization (Figs. 2A and S1A). There is often a nuclear, cell cycle dependent localization of NM23-H1 due to interaction with proteins that act as traffickers, and may also include the viral oncoproteins encoded by Epstein Bar Virus EBNA1 and EBNA3C, and the KSHV encoded protein LANA^12–14^. The p110α catalytic subunit mostly localized to the cytoplasm and following interaction with the p85α regulatory subunit can localize to the plasma membrane as the active kinase^15^. The MDA-MB-435 cell line expressing both Myc-p110α and HA-NM23-H1 showed overlapping signals at the cell membrane, which is consistent with localization of the PI3K complex at the cytoplasmic side of the cell membrane (Figs. 2A and S1A). The NM23-H1 mutants showed similar distribution patterns as the WT isoform. However, Z-sections and the colocalization coefficient (R) analyses did not show any dominant overlap between the NM23-H1 mutant proteins and p110α (Fig. 2A, B). These data further support our conclusions as demonstrated by binding analysis, and confocal microscopy the mutated isoforms of NM23-H1 do not interact with p110α. Therefore, we used the mutated isoforms of NM23-H1 as negative controls for further biochemical and functional studies that will be investigated to elucidated the specific consequence of the interaction between NM23-H1 WT and the p110α catalytic subunit.

### NM23-H1 expression negatively correlated with Akt phosphorylation

**Fig. 3:**
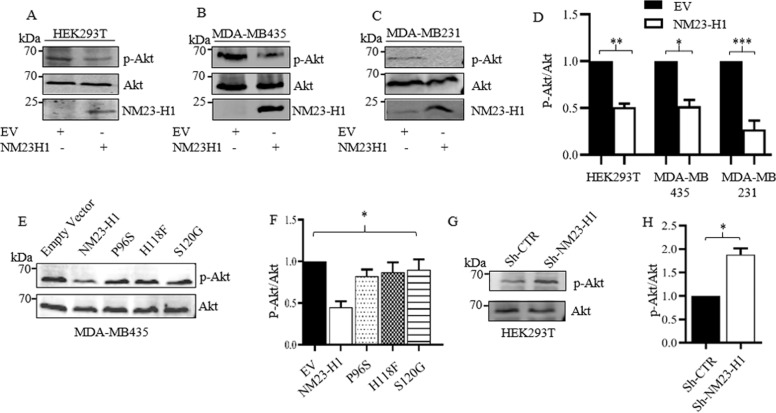
Cells expressing NM23-H1 show a reduction in p-Akt levels. **A**–**C** Phosphorylated Akt was evaluated by immunoblot densitometry analysis and the normalized expression relative to total Akt was represented in the bar graph adjacent to the immunoblot (**D**). Western blot analysis on cell lysate from MDA-MB-435 expressing EV (empty vector), and the mutant proteins P96S, H118F, S120G results are shown in **E**. The Akt phosphorylated levels were normalized with total Akt protein levels (**F**). **G**, **H** Western blot analysis on cell lysates from HEK293T cells knock down for *NM23-H1* expression using Sh-NM23-H1 (Short hairpin) showed increased levels of p-Akt. Fluorescent secondary antibodies were used, and quantification performed using the LI-COR Image Quant system and *T*-test statistical analyses were performed. *N* = 3. (*p < 0.05).

The p110α kinase is involved in production of PI3P from phosphatidylinositol 4,5-biphosphate (or PI2P) that acts as a docking site for proteins containing the Pleckstrin homology (PH) domain. An example is the Akt protein, which is the most characterized protein that interacts with either PI2P or PI3P^8^. This interaction leads to Akt localization at the cell membrane, and allows Akt to be activated by the 3-phosphoinositide-dependent protein kinase-1 (PDK1) and PDK2^16–19^. To confirm a possible role of NM23-H1 in modulating p110α kinase activity which results in Akt phosphorylation (p-Akt), Western blot analyses of cell lysates from HEK293T, MDA-MB-435, and MDA-MB-231 cell lines stably expressing *NM23-H1* were performed (Fig. 3A–C). All cell lines analyzed showed a significant reduction in p-Akt of approximately 2 to 3.5-fold in HEK293T, MDA-MB-435, and MDA-MB-231 (Fig. 3D). Western blot analyses of cell lysates from MDA-MB-435 expressing the mutant proteins P96S, H118F, S120G were also performed (Fig. 3E). The mutant proteins P96S, H118F, and S120G expression did not lead to a reduction in p-Akt levels (Fig. 3F). To determine if *NM23-H1* knock down would lead to a decrease in the negative regulation of p110α which impacts p-Akt levels, Western blot analyses on cell lysates from HEK293T stably knockdown for *NM23-H1* expression was performed (Fig. 3G). The effects of the short hairpin on *NM23-H1* expression are shown in Fig. S2A–C. The results as shown in Fig. 3H, demonstrated that knock down of NM23-H1 led to an approximately 2-fold increase in the level of p-Akt. This data showed a negative correlation between NM23-H1 expression and p-Akt suggesting that the NM23-H1/p110α interaction can lead to a negative regulation of p110α kinase activity, which can compromise the localization of Akt at the cell membrane, and thus influencing the initiation of biochemical events that activate the signaling of the PI3K pathway.

### NM23-H1 can negatively regulate growth factor-mediated PI3K activation

**Fig. 4:**
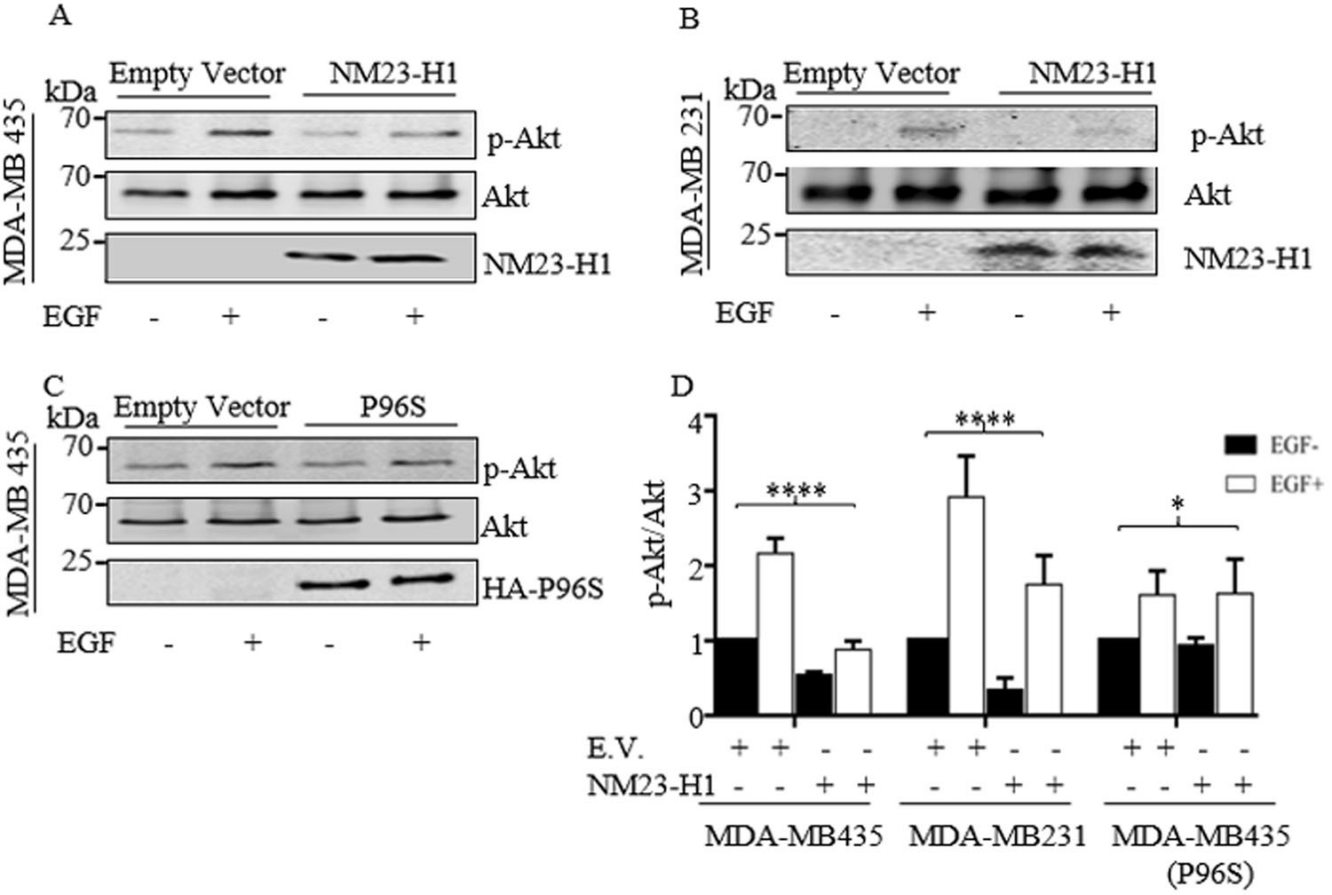
NM23-H1 expression inhibit Akt phosphorylation upon EGF treatment of melanoma and TNBC (triple negative breast cancer) cells. MDA-MB-435 and MDA-MB-231 expressing Empty vector, NM23-H1 and MDA-MB-435 expressing the P96S mutant isoform were starved for 16 h and treated with EGF (20 ng/ml) or vehicle for 10 min. The p-Akt protein was evaluated by immunoblot densitometry analysis on MDA-MB-435 (**A**), MDA-MB-231 (**B**), and MDA-MB-435 P96S (**C**) and the expression were normalized relative to total Akt and represented in the bar graph (**D**). Fluorescent secondary antibodies were used, and quantitation was performed using a LI-COR system. *T*-test statistical analysis was performed. *n* = 3. (*p < 0.05; ****p < 0.0001).

The PI3K-Akt axis is regulated by different stimuli, and growth factor activation of the tyrosine kinase receptor is one of the main mechanisms identified^20^. For example, the epidermal growth factor receptor (EGFR) can activate PI3K signaling^21^. Furthermore, EGF-stimulated induction of actin barbed ends, as well as lamellipodia extensions specifically require the p85/p110α complex in breast cancer cells^22^. Therefore, EGF was chosen as an activator of the PI3K pathway due to its ability to activate cell motility through activation of the PI3K pathway. To demonstrate if NM23-H1 can downregulate p-Akt upon EGF stimulation, Western blot analyses of cell lysates from cell lines MDA-MB-435 as well as MDA-MB-231 expressing *NM23-H1* treated with EGF was performed (Fig. 4A, B). The results showed a reduction in p-Akt levels when *NM23H1* was expressed in MDA-MB-435 and in MDA-MB-231 (Fig. 4D). To explore further if this reduction was due to NM23-H1 expression, the same experiment was performed using the P96S mutant isoform, which does not bind p110α (Fig. 4C). As expected, no significant downregulation of p-Akt levels was seen when compared to control vector (Fig. 4D). These results suggest that NM23-H1 interaction with p110α is involved in the modulation of its downstream kinase activity as seen by a loss in levels of Akt phosphorylation.

### The p110α catalytic subunit increases the motility of human cancer cells in vitro

**Fig. 5:**
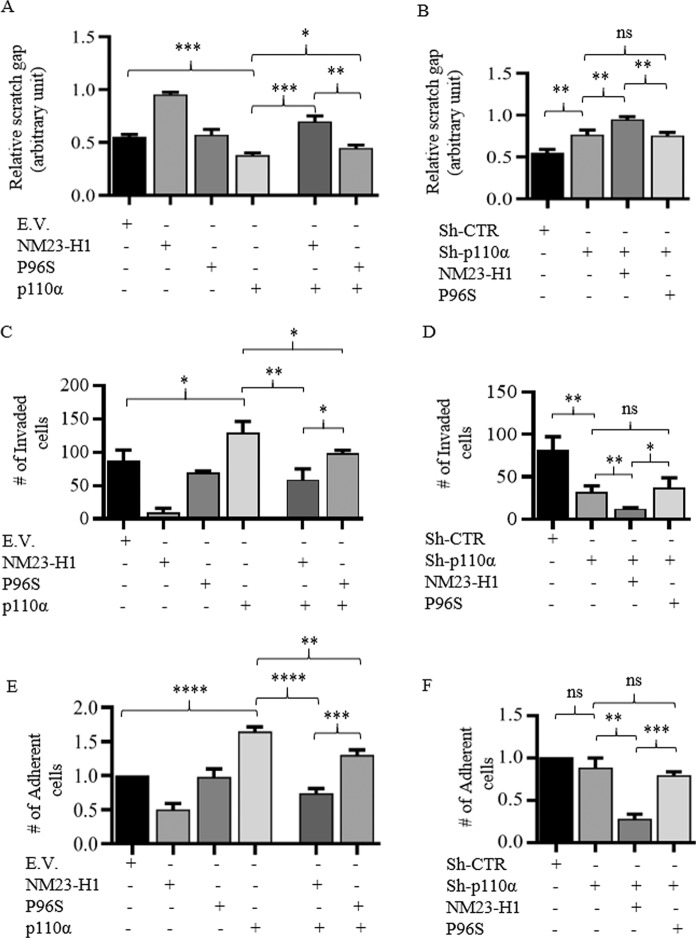
The p110α subunit of PI3K increases motility, invasion, and adhesion of MDA-MB435 cells and nullifies the activity of NM23-H1. **A**, **B** Quantitative results of Wound healing assays were performed by measuring the surface of the scratch. **C**, **D** Quantitative results of Matrigel invasion assays. Quantitative analyses were performed by counting the number of migrated cells using 2% FBS as chemoattractant. **E**, **F** Quantitative results of adhesion assays. Quantitative analyses were performed by counting the number of adherent cells washing, fixing with Methanol, and staining with 0.05% Crystal violet. *T*-test statistical analyses were performed. *n* = 3. (*p < 0.05; **p < 0.01; ***p < 0.001; ns not statistically significant).

Metastasis is a multistage process characterized by dissemination of cancer cells through the activation of many cellular pathways that drives migration, adhesion, and invasion of new tissues. It is the main cause of death for cancer patients^23^. The ability of NM23-H1 to inhibit cellular processes involved in metastasis in tumor cell lines has been widely described over many years^11,24,25^. Moreover, the pro-migratory phenotype of colon cancer cell lines carrying the p110α *gain-of-function* mutation underlines the important role of this protein in promoting metastasis when activated. While the in vitro anti-motility property of NM23-H1 is widely known, we focused our studies on the analysis of these effects when *p110α* and *NM23-H1* are expressed, or during simultaneous silencing expression of *p110α* and *NM23-H1*. The results were then compared to that using the P96S mutant (Fig. 5). The effects of knockdown of p110α expression using short hairpin shRNA are shown in Fig. S3A–C. The results showed that MDA-MB-435 expressing p110α migrated significantly faster than the vector control of 1.45-fold (Figs. 5A and S4A). Co-expression with NM23-H1 resulted in a reduction of 1.85-fold compared to that observed in cells expressing p110α alone (Figs. 5A and S4A). MDA-MB-435 cells expressing the P96S mutant showed a similar trend as vector control. When expressed with p110α the reduction in the wound surface area, that was previously described for cells expressing both NM23-H1 and p110α proteins was not the same (Figs. 5A and S4A). Accordingly, we questioned whether silencing of p110α with a short hairpin RNA (ShRNA) strategy can reduce the ability of the cells to migrate. The results showed that MDA-MB-435-Sh-p110α stable cells resulted in increased wound healing compared to the Sh-CTR (Figs. 5B and S4B). Furthermore, co-expression of the Sh-p110α and NM23-H1 led to a significant reduction in cell motility compared to Sh-CTR (Figs. 5B and S4B). However, expression of the Sh-p110α hairpin with the P96S mutant showed a similar trend to cells containing the Sh-p110α construct, and indicates that expression of the mutated NM23-H1 isoform does not contribute to the reduction of cell motility when compared to the WT counterpart (Figs. 5B and S4B). Invasion is a crucial step in the metastatic process and cancer cells acquire a pro-motility phenotype, and through the extracellular matrix they migrate and reach neighboring tissues. Invasive cancer cells can typically disseminate and metastasize at a second site^26^.

To further support our findings above we applied the same experimental strategy showed for migration in an invasion assay carried out using a Boyden chamber coated with Matrigel solution that mimics the extracellular matrix. MDA-MB-435 p110α expressing cells showed a small increase in the number of invaded cells when compared to vector control (Figs. 5C and S5A). The NM23-H1 expressing cells showed a reduction of approximately 8.6-fold of invaded cells compared to the vector control and were congruent with previous studies, which showed a reduction in the number of invaded cells expressing NM23-H1 (Figs. 5C and S5A). However, no difference was seen when the P96S mutant was expressed in cells compared to vector control (Figs. 5C and S5A). A reduction of invaded cells is showed for the MDA-MB-435 expressing p110α together with NM23-H1 WT, while no significative reduction is showed in the P96S mutant expressing cells (Figs. 5C and S5A). Importantly, silencing of p110α led to a significant reduction in migrated cells of greater than 2.5-fold, which is likely due to the role of the p110α kinase in PI3K signaling and promotion of cell invasion^9,27^ (Figs. 5D and S5B). Simultaneous silencing of p110α and NM23-H1 expression further reduced cell invasion to over 6.5-fold. However, no further reduction was seen in MDA-MB-435 cells expressing the P96S mutant and the Sh-p110α (Figs. 5D and S5B).

The ability of cancer cells to spread and metastasize in the host requires upregulation of cell adhesion molecules that play a critical role in this process, as well as cell–cell interaction with endothelial cells, which is critical for invasion in the vascular system. To determine the adhesion property of the stable MDA-MB-435 cell lines generated above, we performed the adhesion assays (Figs. 5E, F and S6A, B). The results showed that MDA-MB-435 expressing p110α had increased adhesion properties. Additionally, in NM23-H1 expressing cells a reduction in adherent cells was observed when compared to the vector control of approximately 2-fold. Furthermore, the P96S mutant expressing cells showed no significant reduction in adherent cell numbers (Figs. 5E and S6A). Expression of both p110α and the P96S mutant led to a mild reduction of the number of invading and adherent cells (Fig. 5C, E).

It has been shown that P96S, as previously reported, leads to a reduction of the active NM23-H1 hexameric complex favoring the formation of a dimeric inactive complex^28^. We, therefore, cannot exclude the possibility that the P96S mutant induced the formation of NM23-H1 hexameric complexes that retained their enzymatic activity. Moreover, the cells expressing both p110α and NM23-H1 showed a greater reduction in the number of adherent cells when compared with cells expressing p110α alone or with the P96S mutant (Figs. 5E and S6A). The MDA-MB-435 Sh-p110α stable cells showed a small reduction in adherent cell numbers. However, when NM23-H1 was expressed with the Sh-p110α, a clear difference in adherent cell numbers was seen at 3.6-fold compared to the MDA-MB-435 Sh-p110α stable cells alone. No significative difference was detected between MDA-MB-435 Sh-p110α alone or when expressed with the P96S mutant (Figs. 5F and S6B). Overall, these results not only support the anti-motility activity of NM23-H1, but also demonstrated that the activity of NM23-H1 was capable of nullifying the pro-motility phenotype of cells with active PI3K signaling.

### NM23-H1 suppresses p110α induction of filopodia structures

**Fig. 6:**
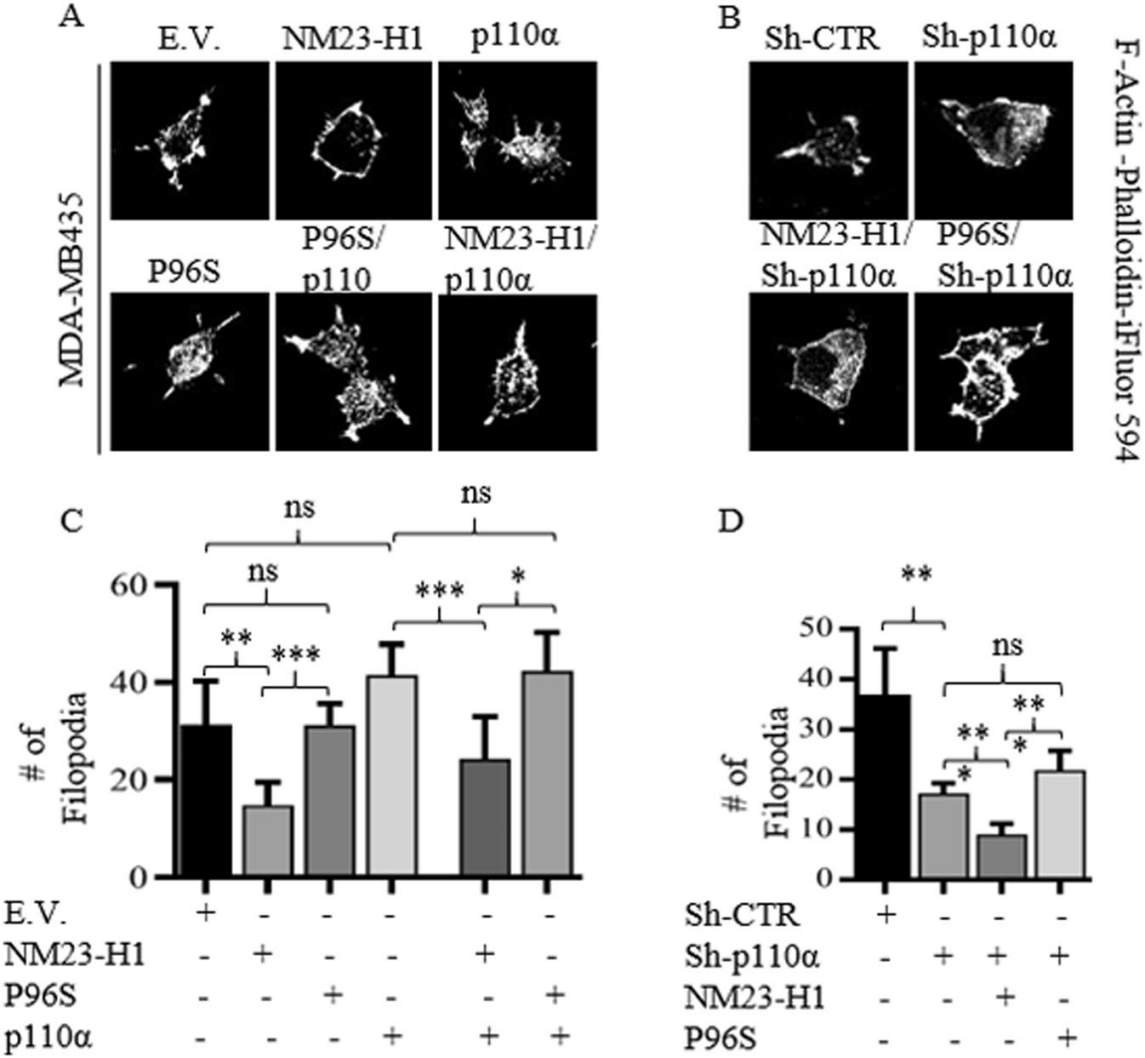
NM23-H1 suppresses the induction of Filopodia structures. **A**, **B** Cell morphology and filopodia structures were analyzed using Fluorochrome-tagged-Phalloidin to stain F-actin. Confocal Images shown were taken at ×60 magnification. Cells were fixed and stained for F-Actin with Fluorochrome-tagged-Phalloidin. **C**, **D** Filopodia structures were manually counted and the average of 5 independent fields is shown. *T*-test statistical analysis was performed. *n* = 3. (*p < 0.05; **p < 0.01; ***p < 0.001; ns not statistically significant).

Cell migration requires the formation of specialized structures that provide cells the ability to move. In particular, protruding structures of the plasmalemma known as Filopodia are fundamental to cellular processes which include migration, invasion, adhesion, angiogenesis, and cell–cell contact^29^. Contextually, it is also known that a specific p110α gain of function point mutation H1047R is responsible for an increased number of Filopodia production, change in cell shape and an increase in metastatic activity^9^. Moreover, we previously showed evidence of direct interaction between NM23-H1 and the small GTPase of the Rho-subfamily Cdc42^30^, a key regulator of different cell functions including cell morphology, migration, endocytosis, and cell cycle progression, and an important role in formation of the extension and maintenance of the actin-rich surface projections filopodia^30^. We therefore asked if p110α expression was able to increase this dynamic process, and if NM23-H1 expression can rescue this process. To examine this process, immunofluorescence analyses on MDA-MB-435 cells stained with the Fluorocrome-tagged Phalloidin and Fascin were performed (Figs. 6A, B and S7A, B). Cells expressing p110α showed a small increase in the number and length of filopodia compared to vector control, while NM23-H1 expressing cells showed a 2-fold reduction in filopodia formation and length when compared to vector control (Figs. 6C and S7A, C). No significant difference was detected for the MDA-MB-435 cells expressing the P96S mutant compared to vector control (Figs. 6C and S7A, C). Cells expressing p110α and NM23-H1 WT showed a decreased number and length of filopodia of 1.7-fold and 2-fold, respectively, when compared with p110α alone. However, when expressed with the P96S mutant no reduction was observed (Figs. 6C and S7A, C). Silencing of p110α affected filopodia formation and length by a decrease of 2-fold and 1.85-fold, respectively, when compared to the vector control. A marked reduction in filopodia numbers and length was observed in cells expressing WT NM23-H1 and Sh-p110α by almost 2-fold and 2.65-fold, respectively (Figs. 6D and S7B, D). There was no significant difference in the number and length of filopodia in cells expressing the P96S mutant when expressed with Sh-p110α (Figs. 6D and S7B, D). Western blot analyses to detect Fascin expression were consistent with confocal analysis of filopodia structures (Fig. S7E–H). The data suggests that restoring NM23-H1 expression in metastatic cell lines will lead to a reduction in pro-motility structures, probably through its negative effects on the PI3K-AKT axis.

### NM23-H1 impairs clonogenicity induced by p110α

**Fig. 7:**
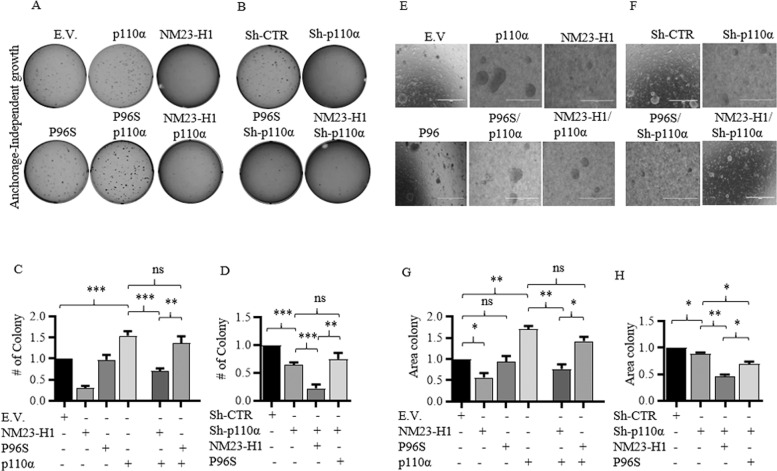
Expression of NM23-H1 with p110α impairs the clonogenicity and anchorage-independent growth of MDA-MB-435 cells. **A**, **B** MDA-MB-435 clones were tested for anchorage-independent growth in soft agar assays. Cells were seeded at low density (1 × 10³). After 14 days cells were stained with 0.05% Crystal Violet O.N. The number (**C**, **D**) and size of colony (**E**–**H**) were counted and represented by a bar graph (**G**, **H**). *T*-test statistical analysis was performed. *n* = 3. (*p < 0.05; **p < 0.01; ***p < 0.001; ns not statistically significant).

The p110α kinase has been implicated in growth and proliferation of tumor cells and targeting p110α activity markedly impairs proliferation and survival of tumor cells^31^. Moreover, other studies describe a striking reduction in proliferation, survival and anchorage-independent growth in the NM23-H1 expressing tumor cell lines^32,33^. To determine whether *NM23-H1* expression can impair p110α-induced proliferation an anchorage-dependent (Fig. S8A–D) and independent growth assays were performed (Fig. 7A–D). The results strongly suggested that while MDA-MB-435 cells expressing p110α showed an increased number of colonies compared to control vector (Fig. S8A, C), no significant differences were detected in MDA-MB-435 cells expressing NM23-H1 WT or P96S mutant (Fig. S8, C). Expression of NM23-H1 and p110α led to a reduction in number of colonies compared to the cells expressing only p110α, of about 1.56-fold. However, the expression NM23-H1 was able to counteract the proliferative effects of p110α (Fig. S8A, C). The MDA-MB-435 Sh-p110α cells did not show a clear reduction in the number of colonies which may be due to a switch in PI3K activity to the p110β isoform, a strategy previously described when p110α expression was impaired by RNA silencing^34^ (Fig. S8B, D). Surprisingly, simultaneous expression of Sh-p110α and NM23-H1 drastically reduced the number of colonies by greater than 3-fold compared to vector control. This was not seen with the P96S mutant in Sh-p110α knockdown cells (Fig. S8B, D).

Anchorage-independent growth was also performed, and the relative size of the colonies was analyzed (Fig. 7). In vitro analysis of anchorage-independent growth of tumor cells is linked to the aggressiveness and metastatic potential of cancer cells^35^. Similar to the previous assay an increase in colony numbers were reported in MDA-MB-435 cell expressing p110α to about 1.4-fold while MDA-MB-435 cells expressing NM23-H1 showed a reduction in colony numbers greater than 3-fold when compared to vector control (Fig. 7A, C). No significant differences were detected between vector control and cells expressing the P96S mutant (Fig. 7A, C). Expression of p110α and NM23-H1 led to a reduction in the number of colonies by 2-fold compared to cells expressing p110α alone. However, there was no substantial difference in colony numbers in cells expressing the P96S mutant and p110α (Fig. 7A, C). A reduction of colony numbers was seen for MDA-MB-435 Sh-p110α cells when compared to the vector control, while Sh-p110α cells expressing the NM23-H1 WT or P96S mutant showed a reduction of colonies by 4.4 and 1.3-fold, respectively (Fig. 7B, D). Colony size analyses showed results were consistent with the reduction of the colony number shown (Fig. 7E–H). These results support our hypothesis that the inhibitory activity shown in the anchorage-dependent and independent-growth assays were due to the ability of NM23-H1 WT to neutralize the activity of p110α in cancer cells.

## Discussion

The PI3K pathway is a hallmark of cancer, and deregulation of the p110α kinase is sufficient for cellular transformation or cancer progression. NM23-H1 suppression of cell migration has been observed in different cancers and a large body of evidence gathered over decades highlight the extensive network of protein–protein interactions, and their role in inhibiting cell motility. In this study, we show the interaction between two proteins with opposite activities as they relate cancer progression. Data from our GST-pull down, and Co-IP assays supports this interaction. Further, analysis of the interaction with the p85α regulatory subunit showed that the interaction was specifically with the catalytic subunit and not the p85α subunit of PI3K protein complex. This interaction was localized at the plasma membrane, where p110α is known to function as an active component of the PI3K protein complex. Analysis of the phosphorylated Akt showed a strong reduction in activation of the PI3K–AKT axis, and also occurs when the pathway is activated by a strong activator such as EGF. Furthermore, downregulation of *NM23-H1* levels led to an increase in phosphorylated Akt, which supports our hypothesis that NM23-H1 can affect this axis. Previous studies have also shown that cell lines knockdown or with increased *NM23-H1* expression had changes in their Akt phosphorylation levels^11,25,36^. Previous studies have shown a negative correlation between *NM23-H1* expression and activation of the PI3K pathway. Low NM23-H1 and high Akt and p-Akt expression was observed in ovarian serous adenocarcinoma and ovarian clear cell adenocarcinoma^36^. Additionally, expression of *NM23-H1* negatively correlated with tumor stage, grade and lymph node metastasis, whereas the expression of Akt/p-Akt was positively correlated with these clinic factors^36^. However, the molecular mechanisms behind these changes have not been previously explored.

The biochemical and functional characterization of the mutated isoforms of NM23-H1 (P96S, H118F and S120G) linked the antimotility activity of NM23-H1 to its different enzymatic activities^37^. Nonetheless, the biochemical analysis of the mutants also showed a structural instability which is associated with reduction of the hexameric complex (typical of the NDPK family) in favor of catalytically inactive dimers^28^. To date, the main mechanism by which NM23-H1 acts as an anti-metastatic protein is still unclear. Analysis of the mutated P96S, H118F, S120G isoforms showed no detected evidence of protein–protein interaction with p110α, although they preserved the same subcellular localization. Expression of these mutants did not induce any obvious downregulation of Akt phosphorylation, suggesting that the mutants are not able to inhibit the PI3K-AKT axis as observed with the NM23-H1 WT. The data therefore suggested that interaction between these two proteins had a clear functional role. We focused on the concomitant expression of p110α and *NM23-H1* and compared the data obtained with the same experimental setting with the P96S mutant. Consistent with the role of p110α in cancer progression, we showed an increase in motility and invasiveness as well as adhesion in MDA-MB-435 expressing p110α. This activity decreased when *NM23-H1* was also expressed and was abrogated when *NM23-H1* was expressed in MDA-MB-435 cells that were knockdown for p110α. These results support a role for p110α activation as a major stimulus for cancer progression, and that restoring *NM23-H1* expression can effectively neutralizes its pro-metastatic activity.

The NM23-H1 interactome also extends to proteins directly involved in actin remodeling with a key role in regulation of cell motility^24,30^. Furthermore, an increased number of filopodia in Human colon cancer cells HCT116 positive for the p110α *gain of function* point mutation H1047R has been previously described^9^. The analysis of the structures associated with cell motility provides an important clues as regards to the dynamic processes associated with actin remodeling in the context of cell migration^38^. Our analysis of filopodia structures showed an increase in the number of filopodia when p110α was expressed alone or with the P96S mutant, while we did not observe this increase when p110α was expressed along with NM23-H1 WT suggesting that NM23-H1 was necessary to negatively regulate the formation of filopodia. When *NM23-H1* expression was restored the results aligned with previous studies which showed that expression of *NM23-H1* affected clonogenicity in an anchorage independent growth assay^39^. Expression of both NM23-H1 and p110α led to a reduction in the clonogenicity induced by p110α expression, and this reduction was evident in MDA-MB-435 Sh-p110α but expressing *NM23-H1*. Notably, we did not see a similar trend when the P96S mutant was expressed.

To comprehensively describe the role of NM23-H1 in regulation of the metastatic process is still very difficult, due to the numerous biological processes associated this protein. However, characterization of this interaction may be fundamental to our understanding of previously described mechanisms including the phosphatidylinositol pathway^11,25,36^. Future studies will focus on the characterization of this interaction from a biochemical and structural perspective. A translational approach will also be interesting as it was previously shown that treatment of MDA-MB-231 with Medroxyprogesterone acetate increases the expression levels of *NM23-H1* with reduction of metastasis formation^40^. Therefore, a strategy which involves both a biochemical and translational approach will likely expose novel areas for new therapies that target the PI3K complex using small molecule inhibitors, and may prove a promising anti-metastatic tumor strategy^41^.

## Materials and methods

### Cells and antibodies

NM23-H1 antibody (sc-514515) and p110α antibody (sc-293172) used for Western blot, Co-IP and Confocal Microscopy were purchased from Santa Cruz Biotechnology, Inc. (Santa Cruz, CA). p-AKT1 (Ser 473), AKT1 (sc-1618), GAPDH (sc-47724), and GFP (sc-53882) were obtained from Santa Cruz Biotechnology, Inc (Santa Cruz, CA). Mouse anti-Myc (9E10) and mouse anti-HA (12CA5) were prepared in our lab from the hybridoma cells. Human anti-Fascin (clone 833223) was purchased from Novus Biological. MDA-MB-435 (Melanoma cell line), MDA-MB-231 (TNBC, Triple negative breast cancer) and HEK-293T (human embryonic kidney cell line) were provided by Professor Jon Aster (Brigham and Woman’s Hospital, Boston, MA). MDA-MB435, MDA-MB231 and HEK-293T were maintained in Dulbecco’s modified Eagle’s medium (DMEM; Gibco) supplemented with 5% Bovine Growth Serum (BGS, HyClone Bovine Growth Serum).

### Transfection, co-immunoprecipitation, and Western blotting

HEK293T, MDA-MB-435, and MDA-MB-231 cells were transfected with jetPRIME (Polyplus Transfection, Illkirch, France) according to the manufacturer’s instructions. Co-IP and Western blotting were performed as described previously^42^.

### Plasmid constructs

pCMV-NM23-H1, pCMV-NM23-H1 (P96S), pCMV-NM23-H1 (H118F), and pCMV-NM23-H1 (S120G) were obtained from Dr. Patricia Steeg (NIH, Bethesda, MD). NM23-H1 mutants P96S, H118F, S120G were cloned into the pCDNA HA-tagged vector at the BamHI-EcoRI sites. Wild type NM23-H1 was cloned into pEGFP, pA3M, pCDNA-HA, and pET-28a vectors at the BamHI-NotI sites using the primers CGGATCCGGAAGGAACCATGGC and CTGTCATTCATAGATCCAGTTCTGAG, the pSG5-p110α vector was obtained from Dr. Downward (Francis Crick Institute, London, UK). p110α was cloned in pGex-6p1 vector at BamHI-XhoI sites. cDNA for individual domains of p110α (p85BD, RBD, C2, helical, and catalytic domain) were amplified by PCR using the following primers: p85BD, CGGGATCCATGCCTCCAAGACCATCA and CGGCGGCCGCACGGTTGCCTACTGG; RBD, GCGGATCCACCATGGAAGAAAAGATC and CGGCGGCCGCGCTTTCTTTAGCCATC; C2 domain, GCGGATCCACCATGCTCTATTCTCAA and CGGCGGCCGCTACCACACTGCTGAA; helical domain, GCGGATCCACCATGAAGTTTCCAGAT and CGGCGGCCGCCCCACATGCACGGCA; and catalytic domain, GCGGATCCACCATGTATCTGAAG and CGGCGGCCGCTCAGTTCAAAGCATG. PCR products of each domain were subcloned into Not1 and BamH1 sites of pGex-6p1 vector.

### Lentiviral production and infection

Sh-RNAs targeted p110α and NM23-H1 were constructed by annealing two pairs of primers. The primers are: 5′tcgagtgctgttgacagtgagcgaCCAGATGTATTGCTTGGTAAAtagtgaagccacagatgtaTTTACCAAGCAATACATCTGGgtgcctactgcctcggaa3′(Shp110α.1); 5′cgagtgctgttgacagtgagcgaGCATTAGAATTTACAGCAAGAtagtgaagccacagatgtaTCTTGCTGTAAATTCTAATGCgtgcctactgcctcggaa3′(Shp110α.2); 5′tcgagtgctgttgacagtgagcgaGTCTGAAGTTTCTGCAGGCTTtagtgaagccacagatgtaAAGCCTGCAGAAACTTCAGACgtgcctactgcctcggaa3′(ShNM23-H1.1); tcgagtgctgttgacagtgagcgaCGGCCTGGTGAAATACATGCAtagtgaagccacagatgtaTGCATGTATTTCACCAGGCCG gtgcctactgcctcggaa (ShNM23-H1.2).

The cloning strategy, Lentivirus production and transduction have been described previously^43,44^.

### Wound healing assay

MDA-MB-435 cells were seeded at a density of 1–5 × 10^5^ cells/well in 12-well culture plates and allowed to form a confluent monolayer. The layer of cells was scraped with a 20–200 μL micropipette tip to create a wound of ~1 mm width. Images of the wounds were monitored under a phase-contrast microscope at ×100 magnification at T0h and T24h. All values were expressed as mean ± SEM.

### Adhesion assay

Ninety-six-well plate were coated with 10 μg/mL mouse collagen IV (BD Biosciences inc., San Jose, CA) for 30 min at 37 °C. Wells were then washed with 1 mg/mL BSA DMEM and blocked for 1 h with 5 mg/mL BSA DMEM at 37 °C, 5% CO_2_. Subsequently, cells were seeded into wells (2 × 10^4^ per well) and allowed to adhere for 30 min. Wells were washed four times with 1 mg/mL BSAin DMEM and adherent cells were fixed, stained with crystal violet, and dried overnight. Cells were then counted with ImageJ software (https://imagej.nih.gov/ij/, 1997–2018).

### Transwell invasion, colony formation, and soft agar assay

The Matrigel invasion assays were done using BD Biocoat Matrigel-coated invasion chambers (BD Biosciences inc., San Jose, CA). FBS (2%) was applied as a chemoattractant. Cells that had invaded through Matrigel to the other side of the 4-μm porous membrane were fixed and stained with crystal violet. Membranes were then affixed to glass slides and examined microscopically at ×100 magnification. Cells in a representative region of each membrane were counted with ImageJ. MDA-MB-435 cell line was assayed in triplicate chambers and in duplicate experiments. The colony formation and soft agar assays were performed as described previously^42^.

### Confocal microscopy

For immunofluorescence, cells were seeded on glass coverslips in 24-well plates before transfection. After treatment, cells were fixed with 4% paraformaldehyde at 4 °C for 60 min and permeated with 0.2% Triton X-100 in PBS for 10 min. The nuclei were visualized by staining with DAPI for 2 min. Images were acquired using a Fluoview FV300 confocal microscope and Fluoview software was used for image analysis.

### RNA isolation and real-time PCR

The total RNA extraction and quantitative real-time PCR analysis were performed as described previously^42^. The used primers for NM23-H1 and p110α expression detection are: p110α forward 5′-CTGCAGTTCAACAGCCACAC-3′; p110α reverse 5′-ACAGGTCAATGGCTGCATCA-3′; NM23-H1 forward 5′-TCTGGCCTTTTCTTCACAGC-3′; NM23-H1 reverse 5′-GCTCCCGCTTTGTGTTTATT-3′.

### Statistical analysis

All experiments in the present study were performed at least two times and similar results were obtained. The statistical analysis were performed using the “two-tailed paired Student’s *T*-test”. The program GraphPad Prism 8.0.2 software was used. A value of at least *p* < 0.05 was considered statistically significant. (*p < 0.05; **p < 0.01; ***p < 0.001; ns not statistically significant).

## Supplementary information

Supplemental Figures Legends

Supplemental Figure 1

Supplemental Figure 2

Supplemental Figure 3

Supplemental Figure 4

Supplemental Figure 5

Supplemental Figure 6

Supplemental Figure 7

Supplemental Figure 8
