# Supplementary material for: The metastasis suppressor protein NM23-H1 modulates the PI3K-AKT axis through interaction with the p110α catalytic subunit
Source: Oncogenesis. 2021 Apr 30;10(4):34. doi: 10.1038/s41389-021-00326-x (PMC8087825; doi:10.1038/s41389-021-00326-x)
Supplement: Supplementary file 1 — Supplemental Figures Legends [file 41389_2021_326_MOESM1_ESM.docx]

**Supplemental Figures Legends**

**
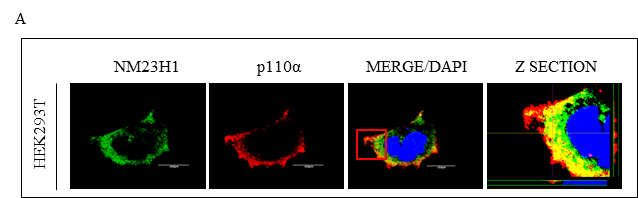
**

**Supplemental Figure 1. p110α co-localizes with NM23‐H1 in HEK293T.** A) Cells were transfected with both HA-NM23‐H1 and Myc-p110α. Cells were fixed in 4% PFA and screened for NM23-H1 and p110α signal using the anti-HA antibody and anti-p110α antibody, respectively. Proteins were detected with anti-HA antibody NM23‐H1 (green), and anti-p110α antibody (red). Nuclei were stained with 4′,6′‐diamidino‐2‐phenylindole (DAPI, blue). Merged images were composed from 3 independently acquired images. Scale bar 10µm.


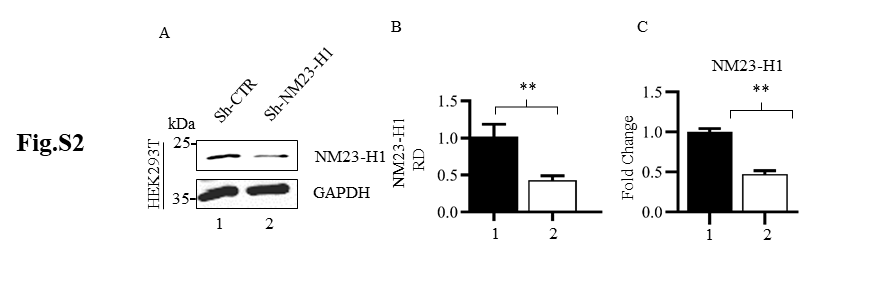


**Supplemental Figure 2. Validation of the knockdown effect of Short hairpin RNA against NM23-H1.** Western Blot and RealTime analysis on cell lysate from MDA-MB-435 cells knockdown for NM23-H1 expression. MDA-MB-435 cell line expressing a short hairpin construct targeting NM23-H1 contains reduced NM23-H1 protein levels as shown by Western Blot for NM23-H1 (A) and quantitated by LICOR Image Quant (B). The mRNA transcripts of NM23-H1 are shown in (C).

**
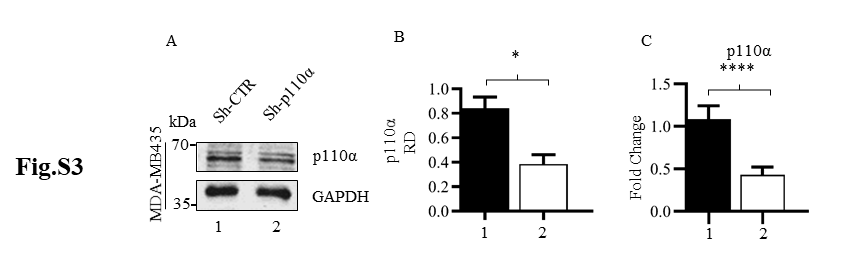
**

**Supplemental Figure 3. Validation of the knockdown effect of Short hairpin RNA against p110α.** Western Blot and RealTime analysis on cell lysate from MDA-MB-435 cells knockdown for p110α expression. MDA-MB-435 cell line expressing a short hairpin construct targeting p110α contains reduced p110α protein levels as shown by Western Blot for p110α (A) and quantitated by LI-COR Image Quant (B). The mRNA transcripts of p110α are shown in (C).


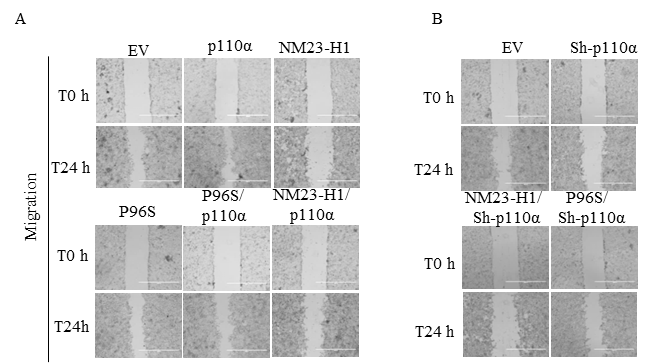


**Supplemental Figure 4. p110α increases motility of MDA-MB-435 cells and NM23-H1 suppresses this activity.** A, B) Representative images of wound healing assay, which was performed by measuring the surface of the scratch at different time points in the same field. The cells expressing p110α, NM23-H1, and P96S (A) and cells knockdown for p110α (B) were seeded and after reaching an optimal confluence scratches were made using a pipette tip through the entire center of the well.

**
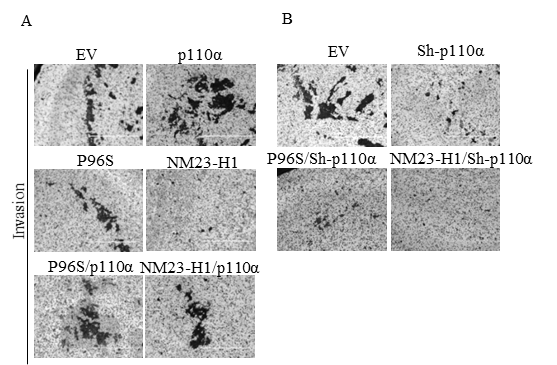
**

**Supplemental Figure 5. p110α increases the invasiveness of MDA-MB435 cells and NM23-H1 inhibits this process.** Representative images (A, B) of Matrigel invasion assay. The cells expressing p110α, NM23-H1, and P96S (A) and cells knockdown for p110α (B) were tested for invasion using Matrigel coated chamber. 2% FBS was used as chemoattractant.


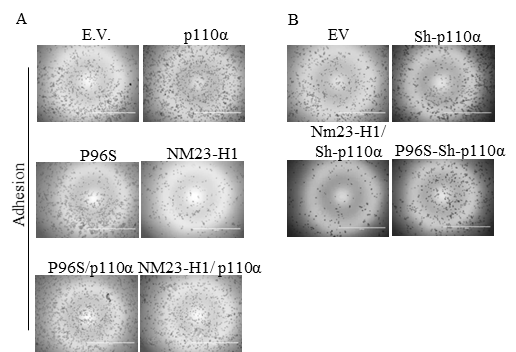


**Supplemental Figure 6. p110α increase adhesion property of MDA-MB-435 cell line and NM23-H1 impair this process.** Representative images (A, B) of adhesion assay. The cells expressing p110α, NM23-H1, and P96S (A) and cells knockdown for p110α (B) were tested for adhesion. The adherent cells were stained with 0.005% Crystal violet after 3 steps of washing and fixed with Methanol.

**
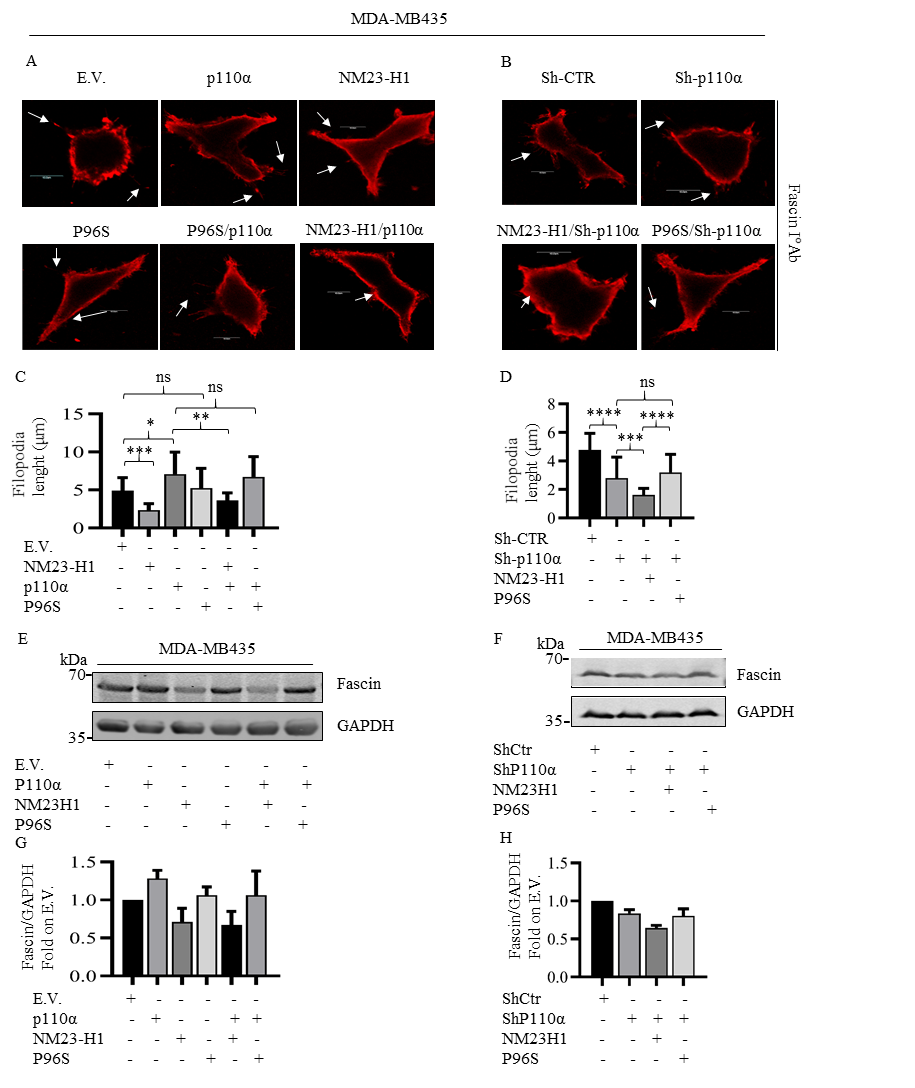
**

**Supplemental Figure 7. NM23-H1 expression leads to a reduction in Filopodia length and protein expression.** A, B) Filopodia structures were analyzed using anti-Fascin antibody. Confocal Images shown were taken at 60x magnification. C, D) Filopodia length were measured using ImageJ-NIH software. MDA-MB-435 cell line expressing NM23-H1 contains reduced Fascin protein levels as shown by Western Blot for Fascin (E, F) and quantitated by LI-COR Image Quant (G, H). T-test statistical analysis was performed. n=2. (* p < 0.05; ** p< 0.01; *** p< 0.001; **** p< 0.0001; ns = Not statistically significant).

**
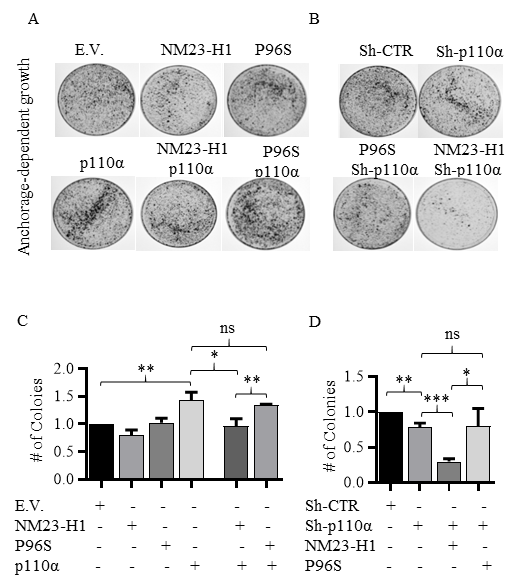
**

**Supplemental Figure 8.**  **NM23H1 and p110α expression impairs clonogenicity of MDA-MB-435 cells in anchorage-dependent growth.** A, B) Clonogenicity of MDA-MB-435 in different expression conditions was measured seeding cells at low density (1x10⁴). After 14 days cells were fixed with 4% PFA and stained with 0.25% Crystal Violet and then counted. Quantitative results are represented by bar graph (C, D). T-test statistical analysis was performed. n=3. (* p < 0.05; ** p< 0.01; *** p< 0.001; ns = Not statistically significant).
