# Supplementary figures and images for: The metastasis suppressor protein NM23-H1 modulates the PI3K-AKT axis through interaction with the p110α catalytic subunit
Source: Oncogenesis. 2021 Apr 30;10(4):34. doi: 10.1038/s41389-021-00326-x (PMC8087825; doi:10.1038/s41389-021-00326-x)

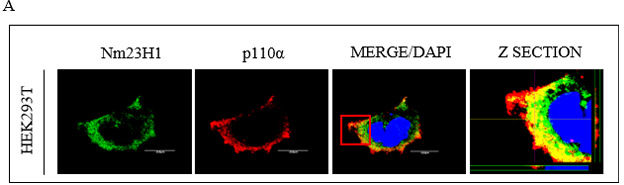

Supplement: Supplementary file 2 — Supplemental Figure 1 [file 41389_2021_326_MOESM2_ESM.tif]

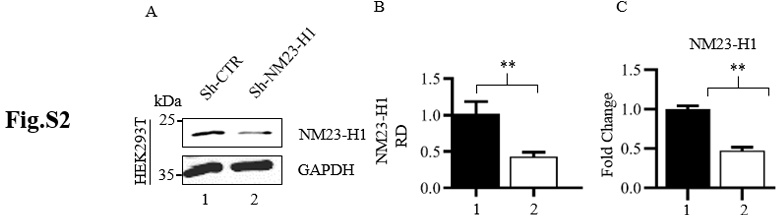

Supplement: Supplementary file 3 — Supplemental Figure 2 [file 41389_2021_326_MOESM3_ESM.tif]

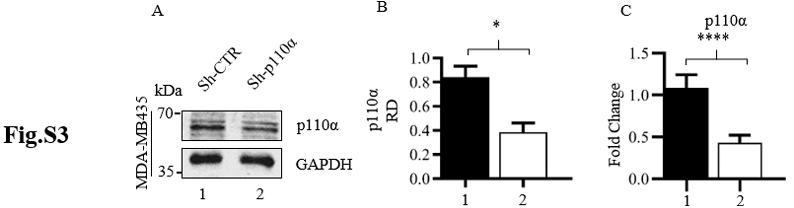

Supplement: Supplementary file 4 — Supplemental Figure 3 [file 41389_2021_326_MOESM4_ESM.tif]

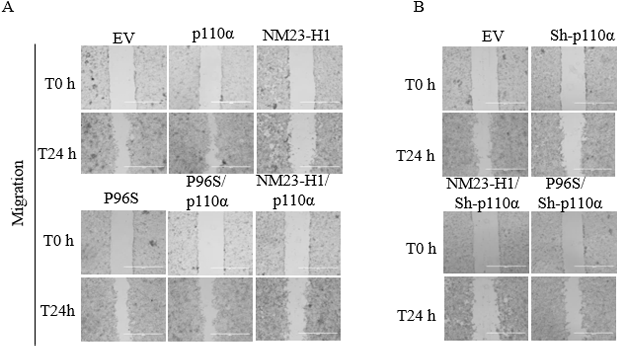

Supplement: Supplementary file 5 — Supplemental Figure 4 [file 41389_2021_326_MOESM5_ESM.tif]

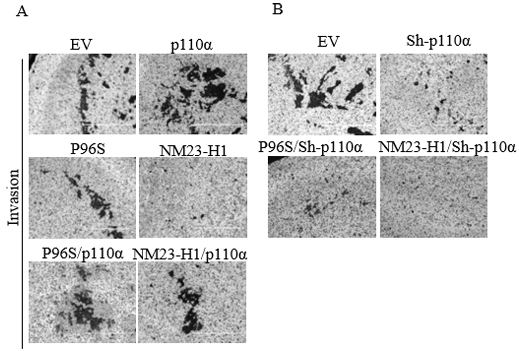

Supplement: Supplementary file 6 — Supplemental Figure 5 [file 41389_2021_326_MOESM6_ESM.tif]

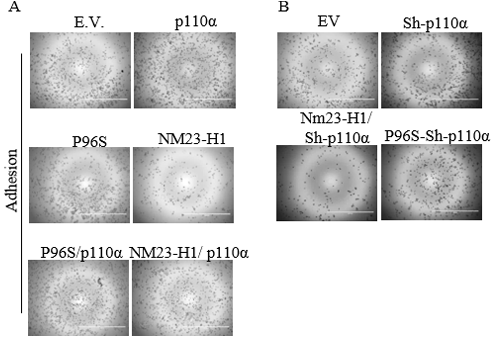

Supplement: Supplementary file 7 — Supplemental Figure 6 [file 41389_2021_326_MOESM7_ESM.tif]

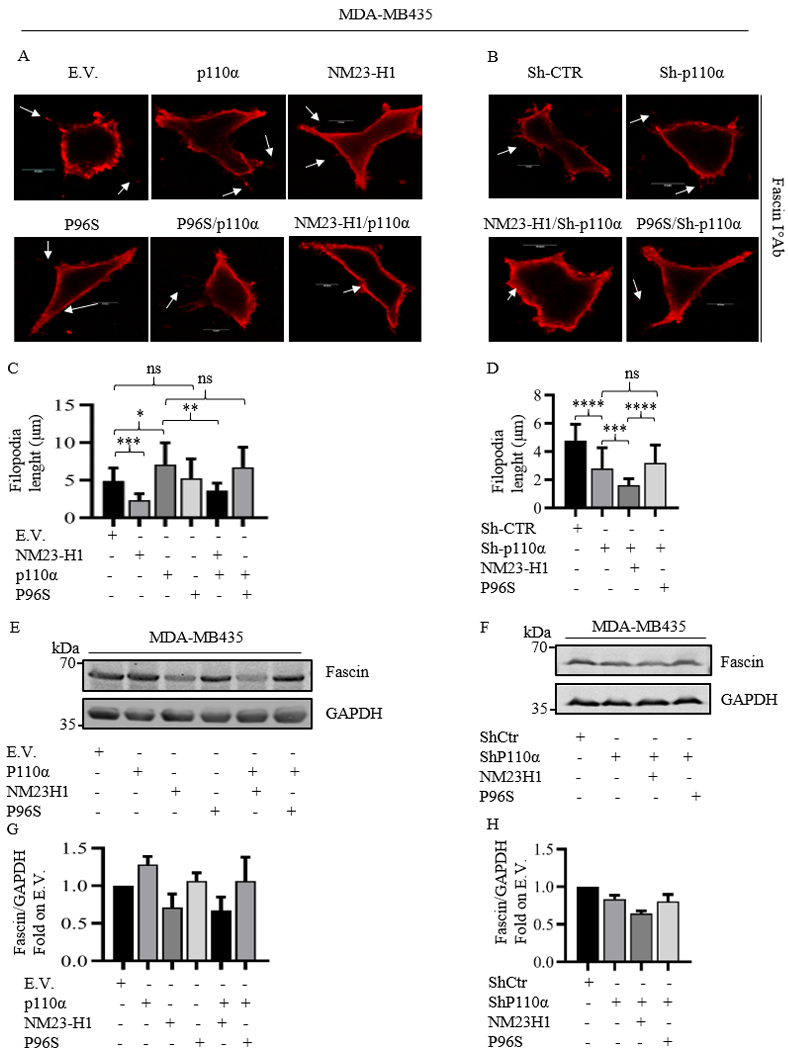

Supplement: Supplementary file 8 — Supplemental Figure 7 [file 41389_2021_326_MOESM8_ESM.tif]

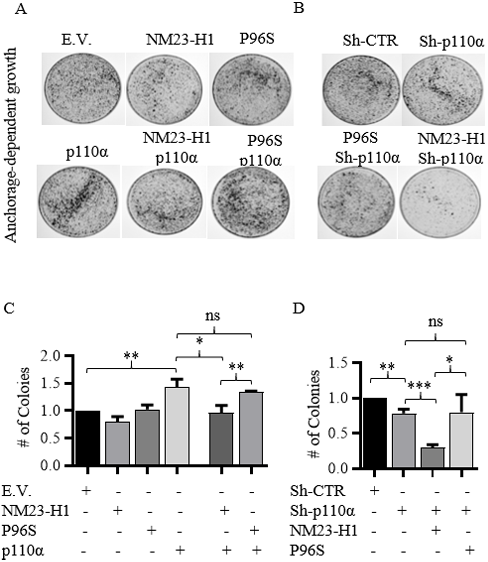

Supplement: Supplementary file 9 — Supplemental Figure 8 [file 41389_2021_326_MOESM9_ESM.tif]
